# Analysis of inter- and intra fractional partial bladder wall movement using implanted fiducial markers

**DOI:** 10.1186/s13014-017-0778-z

**Published:** 2017-03-01

**Authors:** Kentaro Nishioka, Shinichi Shimizu, Nobuo Shinohara, Yoichi M. Ito, Takashige Abe, Satoru Maruyama, Norio Katoh, Rumiko Kinoshita, Takayuki Hashimoto, Naoki Miyamoto, Rikiya Onimaru, Hiroki Shirato

**Affiliations:** 1 Department of Radiation Oncology, Hokkaido University Graduate School of Medicine / School of Medicine, Sapporo, Japan; 2 Department of Renal and Genitourinary Surgery, Hokkaido University Graduate School of Medicine / School of Medicine, Sapporo, Japan; 3 Department of Biostatistics, Hokkaido University Graduate School of Medicine, Sapporo, Japan; 4 Department of Radiation Medicine, Hokkaido University Graduate School of Medicine / School of Medicine, Sapporo, Japan; 5 Department of Radiation Oncology, Hokkaido University Hospital, Sapporo, Japan; 6 Department of Medical Physics, Hokkaido University Graduate School of Medicine / School of Medicine, Sapporo, Japan; 7 Global Station for Quantum Medical Science and Engineering, Global Institution for Collaborative Research and Education (GI-CoRE), Hokkaido University, Sapporo, Japan

**Keywords:** Bladder cancer, Interfractional uncertainty of position, Intrafractional uncertainty of position, Real-time tumor tracking system

## Abstract

**Background:**

Current adaptive and dose escalating radiotherapy for muscle invasive bladder cancer requires knowledge of both inter-fractional and intra-fractional motion of the bladder wall involved. The purpose of this study is to characterize inter- and intra-fractional movement of the partial bladder wall using implanted fiducial markers and a real-time tumor-tracking radiotherapy system.

**Methods:**

Two hundred fifty one sessions with 29 patients were analysed. After maximal transurethral bladder tumor resection and 40 Gy of whole bladder irradiation, up to six gold markers were implanted transurethrally into the bladder wall around the tumor bed and used for positional registration. We compared the systematic and random uncertainty of positions between cranial vs. caudal, left vs. right, and anterior vs. posterior tumor groups. The variance in intrafractional movement and the percentage of sessions where 3 mm and 5 mm or more of intrafractional wall movement occurring at 2, 4, 6, 8, 10, and at more than 10 min until the end of a session were determined.

**Results:**

The cranial and anterior tumor group showed larger interfractional uncertainties in the position than the opposite side tumor group in the CC and AP directions respectively, but these differences did not reach significance. Among the intrafractional uncertainty of position, the cranial and anterior tumor group showed significantly larger systematic uncertainty of position than the groups on the opposite side in the CC direction. The variance of intrafractional movement increased over time; the percentage of sessions where intrafractional wall movement was larger than 3 mm within 2 min of the start of a radiation session or larger than 5 mm within 10 min was less than 5%, but this percentage was increasing further during the session, especially in the cranial and anterior tumor group.

**Conclusions:**

More attention for intrafractional uncertainty of position is required in the treatment of cranial and anterior bladder tumors especially in the CC direction. The optimal internal margins in each direction should be chosen or a precise intrafractional target localization system is required depending on the tumor location and treatment delivery time in the setting of partial bladder radiotherapy.

## Background

Many studies have reported comparable results for trimodality therapy, including transurethral resection of bladder tumors, radiotherapy and chemotherapy of muscle invasive bladder cancers, and standard surgical treatment in selected patients [1–4]. Although a dose–response relationship in bladder cancer patients has been reported [5], dose escalation for the whole bladder presents the risk of global bladder dysfunction (contracted bladder). For this reason, dose-escalated partial bladder radiotherapy has recently been investigated to enable improvement of local control [6–9].

The urinary bladder is a hollow organ which moves mainly due to the urine inflow and it is commonly accepted that the position and volume of the bladder is continually changing. Large differences in urinary inflow rates and initial bladder volumes between individuals have been reported even when patients have received drinking and voiding instructions [10–12]. Therefore, when administering radiotherapy for bladder cancer, adding at least 2 cm margins has been considered to be required to compensate for the uncertainties in size [13–17]. However, this large margin and treatment field may later result in toxicity, especially for the bowels [18–20].

Recently, a number of image guidance technologies such as cone-beam computed tomography (CBCT), ultrasonography, cine-magnetic resonance imaging (MRI), and internal fiducial markers (spherical gold seeds, titanium clips, or lipiodol) have been rigorously assessed [11, 12, 21–24]. In addition, image-guided adaptive radiotherapy is reported to be useful to reduce the CTV to PTV (clinical target volume to planning target volume) margin without reducing the CTV coverage, with a consequent reduction in the dose to surrounding normal tissue and to the volume of the small bowel [25–28]. Although these image guidance and adaptive protocols based on the imaging just before the initiation of radiotherapy are effective to reduce CTV to PTV margins for interfractional bladder wall movements, it is also necessary to pay attention to intrafractional wall movement to prevent subsequent insufficient dose delivery due to the smaller CTV to PTV margins. Based on the pre- and post-treatment CBCT or repeat MRI series, 5–12 mm anisotropic margins have been suggested to cover intrafractional positional uncertainties and the largest movement has been reported to be in the cranial anterior direction [29–31]. The movement of the whole bladder was analysed in these studies, however, the inter- and intra-fractional uncertainty of position of the partial bladder wall are still not clearly established.

Real-time tumor-tracking radiotherapy (RTRT) systems have the advantage that they enable corrections of the target location and also make it possible to observe the location of the target using the fiducial markers during the beam delivery [32]. We have previously reported about intrafractional prostate motion in prostate cancer radiotherapy [33], and have applied the same technique to treat locally advanced bladder cancers with encouraging results [34].

The aim of the study here is to evaluate inter- and intra-fractional positional uncertainty of the partial bladder wall during radiotherapy.

## Methods

### Patients

Thirty-three patients were treated for bladder tumors and administered radiotherapy with a localized boost from 1999 to 2016 with the RTRT system using fiducial markers for the positioning (details specified below). Of these, one patient was excluded because the tumor had massively invaded into the prostate and its motion was expected to be different from that of normal bladders. In addition, 3 other patients who were treated in 1999 were excluded from the analysis because position tracking data had been lost. Finally, 29 patients were included in this retrospective study. Patient particulars, tumor stages, and details of the tumor locations on the bladder wall are shown in Tables 1 and 2. Except for one T3 patient, all of T3 and T4 tumors located in the caudal half of the bladder. By using the RTRT system and the implanted fiducial markers, we are able to see the movements of the part of the bladder wall where the tumor is located; however, we cannot assess the movements of other parts of the bladder wall. As a result we assessed the movement of only one wall segment, that where the tumor was located, for one patient. The patients were divided into eight subgroups based on the primary location of the tumor, according to the 2x2x2 combinations of cranial or caudal, left or right, and anterior or posterior. To compare between cranial vs. caudal bladder wall movement, we defined the cranial tumor group as the four cranial subgroups and the caudal tumor group as the four caudal subgroups. In the same way, we compared between left vs. right and anterior vs. posterior tumor group to assess left vs. right and anterior vs. posterior bladder wall movement.

**Table 1:** Patient characteristics

Median age – years (range)		78 (58–90)
Gender – No.	Male	20
	Female	9
Tumor stage – No. (%)	T2	21 (72%)
	T3	6 (21%)
	T4	2 (7%)

**Table 2:** Tumor locations, stages and subgroups

	Cranial		Caudal	
	Left	Right	Left	Right
Anterior	A: 2 T2:1, T3:1	B: 5 T2:5	E: 2 T2:2	F: 2 T3:1, T4:1
Posterior	C: 3 T2:3	D: 3 T2:3	G: 7 T2:5, T3:1 T4:1	H: 5 T2:2, T3:3

The capital letters (A-H) indicate the name of subgroups and with patient numbers. The breakdown of patients by tumor stage is also shown. The cranial and caudal tumor groups consist of subgroups A, B, C, and D (*n* = 13) and E, F, G, and H (*n* = 16), respectively. In the same way, the anterior and posterior tumor groups consist of subgroups A, B, E, and F (*n* = 11) and C, D, G, and H (*n* = 18), respectively. The left and right tumor groups consist of subgroups A, C, E, and G (*n* = 14) and B, D, F, and H (*n* = 15), respectively

### Treatment

Maximal transurethral resection of the bladder tumors (TUR-BT) was followed by 40 Gy irradiation in 16–20 fractions to the whole bladder with an isotropic margin of 15–20 mm. Implantation of fiducial markers was performed after the delivery of the 40 Gy to minimize the duration between the implantation and the end of the radiotherapy and to avoid loss of markers. One or two markers were transurethrally implanted in the bladder wall of each patient around the primary tumor bed (the TUR-BT scar) before January 2002. Later, from February 2002, the practice was changed, and four to six markers were implanted around the primary tumor bed. Of the 29 patients, 6 patients had been treated before January 2002 and 23 patients after. The interruption of RT to implant the markers was scheduled to be less than 12 days. A localized boost of the primary tumor bed (25 Gy/10 fractions) was given using the RTRT for target positioning. All patients were treated using three-dimensional conformal radiotherapy. We have reported details of the methods and results elsewhere [34].

### Patient data acquisition and treatment planning for the local boost

Computed tomography (CT) images, in 1.25–2 mm slices, of the small pelvis were used for the treatment planning. Until 2004, the CT scanning was preceded by intravesical instillation of 100 ml sterile normal saline to ensure a constant bladder volume. The pre-scanning procedure was changed in 2005: now patients were instructed to avoid urination for 30 min before the CT scanning to allow the bladder to fill with urine. All treatments were administered 30–60 min after the last voiding.

The coordinates of the fiducial markers and the target volumes were determined on a three-dimensional radiation treatment planning (3D-RTP) system using the CT images.

### Positioning procedure using the RTRT system

**Fig. 1:**
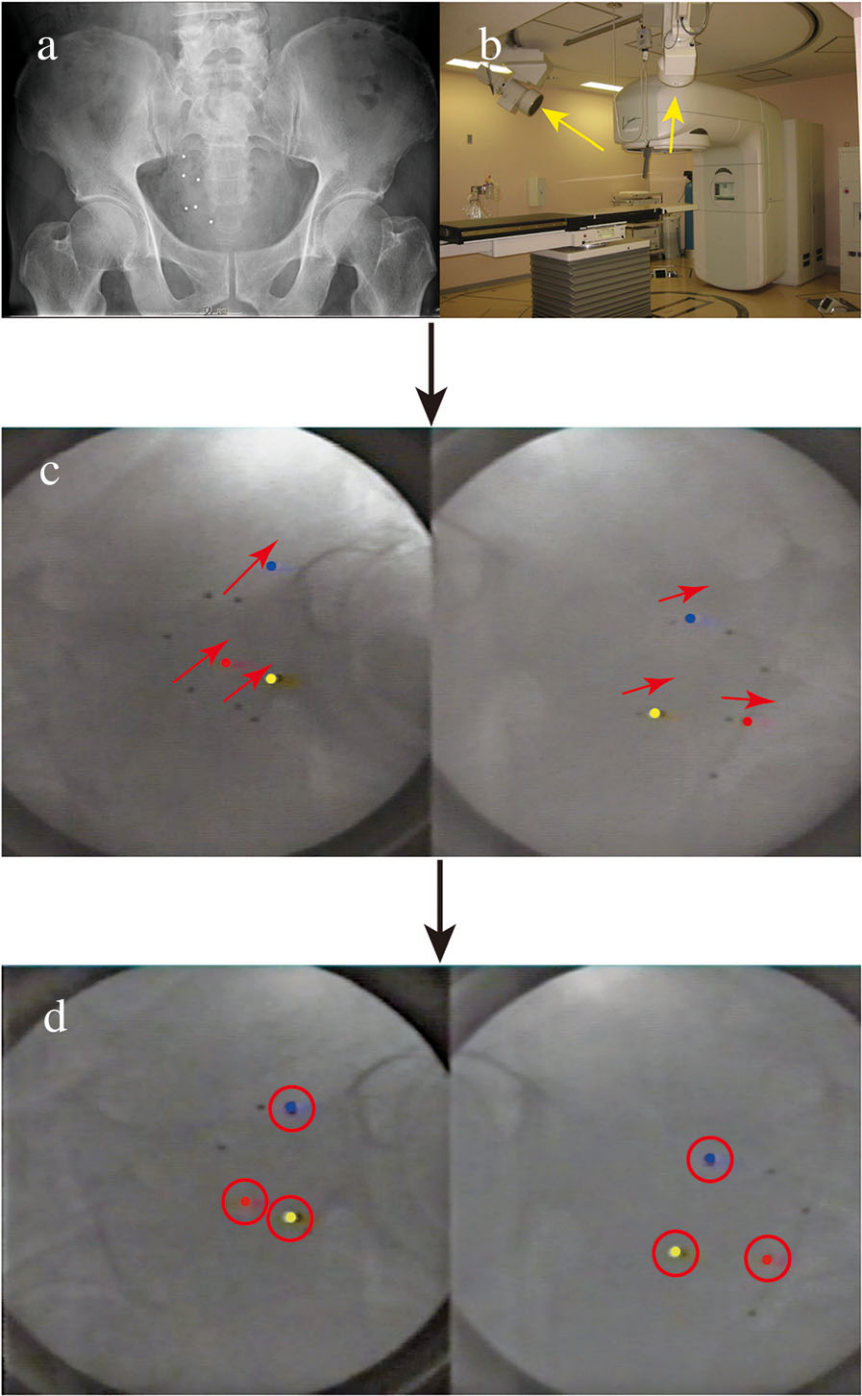
Example of fiducial marker registration. **a** X-ray of patient abdomen with the tumor located in the right bladder wall. This patient had 6 gold markers implanted around the transurethral resection of the bladder tumor (TUR-BT) scar. **b** Arrangement of the fluoroscopic units with the direction of the X-ray beam (arrows). The images were acquired by the two units that are a part of the RTRT system. **c** The colored circles in the fluoroscopic image indicate the gold marker positions at the registration and the black dots represent the actual positions of the gold markers. Up to three markers could be displayed in this system. The red arrow indicates the direction of the required positional correction. **d** Fluoroscopic images after fiducial marker registration. The red circles indicate the fiducial markers placed at the planned position

The RTRT system consists of a linear accelerator, two diagnostic X-ray fluoroscopes in the linear accelerator room, image processing units and an image display unit (originally Mitsubishi; before replacement with one from Varian Medical Japan Co., Tokyo), as reported elsewhere [21]. The actual position of the markers can be visualized on the fluoroscopic image screen during the irradiation. The planned marker position is transferred from the 3D-RTP and superimposed on the display unit screen of the RTRT system. The required distance to correct the position was derived using the RTRT system. After completing the manual setup using skin markings in the supine position, the patient couch was moved so that the markers moved to the planned position (fiducial marker registration) (Fig. 1c and d show an example). For the positional registration, the center of gravity of three markers or the position of one of the markers was used. The center of gravity of three selected markers was used for patients who had more than three markers implanted. For patients who had fewer than three markers implanted, one of the markers identified as that nearest to the isocenter was used. If the positional relationship of the markers was considered to be changed from that on the treatment planning CT, an additional CT scan was performed to identify each marker and confirm the cause of the positonal change. During the treatment, the position of the fiducial markers was continuously observed. If necessary, the treatment was interrupted and the operator could correct the patient position; the threshold used in this study was a 2.0 mm discrepancy from the planned position of the center of gravity of three markers or the position of a single marker in each of the cranial-caudal (CC), left-right (LR) and anterior-posterior (AP) directions. A positive(+) shift represents a translation in the cranial (Cranial +), left (Left+) and anterior (Anterior+) directions.

Of the 29 patients, 28 were treated with 10 irradiation sessions as planned using the RTRT system; one patient wanted, for religious reasons, to avoid the risk of needing a blood transfusion in case of side-effects and the boost radiotherapy for this patient was terminated at 20 Gy/8 fractions; as a result datasets for a total of 288 sessions were obtained. Of these datasets, 37 sessions were excluded because of insufficient records or breaks in the treatment caused by the general condition of the patients involved. Finally, datasets for 251 (87%) sessions were used for the analysis.

### Analysis of wall movements during and between fractions

The marker position at the start of the radiation dose delivery was assigned as the reference position; the pretreatment shifts between skin marker registration and fiducial marker registration were considered as wall movements between fractions (interfractional wall movements), and the shifts of the fiducial markers from the reference position after the start of the treatment were assigned as wall movements during a fraction (intrafractional wall movements).

The total systematic uncertainty of position (Σ) and the overall random uncertainty of position (σ) for both of inter- and intra-fractional bladder wall movements were calculated, and the average of the systematic bladder wall movements of the whole population is the mean overall uncertainty of position (M). The Σ is calculated as the standard deviation of all of the individual systematic uncertainties of position, and the σ is the quadratic average of all the random uncertainties of position. To calculate the intrafractional Σ and σ, we used the maximum wall movement from the reference position at the end of session along each direction not to underestimate intrafractional movement. For instance, if wall movements of 3 mm and 2 mm in the same direction were observed sequentially, the final wall movement would be assigned as 5 mm. On the other hand, if there was a subsequent wall movement of 2 mm in opposite direction to the initial 3 mm movement, the wall movement remained 3 mm even though the final position was located only 1 mm away from the reference position.

In addition to Σ and σ, the variance of the intrafractional movement and the percentage of the sessions with 3 mm and 5 mm or larger intrafractional wall movements occurred at 2, 4, 6, 8, 10, and longer than 10 min until the end of a session were determined to characterise the details of intrafractional motion. The variance of each tumor group was calculated as the mean of all of the individual variances.

We compared the uncertainties of position between cranial vs. caudal, left vs. right, and anterior vs. posterior tumor group in the CC, LR and AP directions, respectively. The mean overall uncertainty of position was compared using an unpaired Welch’s t-test, the systematic uncertainty of position using Levene’s test for equality of variance and the random uncertainty of position using the Mann-Whitney test. Because three separate hypotheses (cranial vs. caudal, left vs. right and anterior vs. posterior) were tested, the significance level was set at 0.05 / 3 after the Bonferroni correction. All statistical analyses were performed using JMP Pro 12.0.1 (SAS Institute, Cary, NC, USA).

## Results

**Fig. 2:**
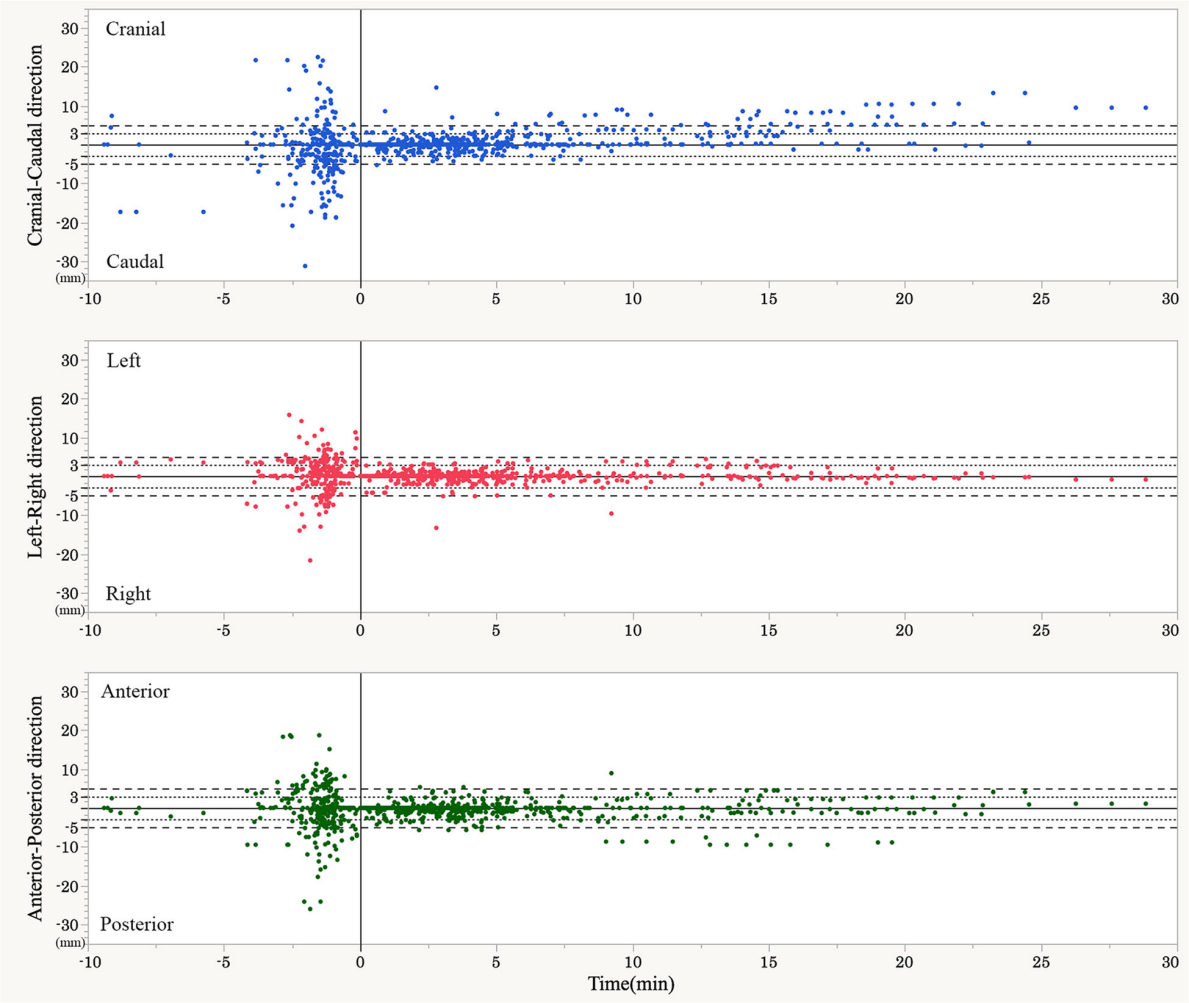
The magnitudes of wall movements in the primary six directions plotted vs time before and after the initial setup at the start of each session. The horizontal axis shows the time in minutes from the initial setup. The vertical axis represents the magnitude of the wall movements in millimeters. The magnitude in the CC, LR, and AP directions in the different graphs are plotted at the same scales. The dotted transverse lines indicate the range of +/− 3 mm wall movements and the broken lines indicate the range of +/− 5 mm wall movements

The ranges of the interfractional wall movements (5% and 95% quantiles) were −31.3 to 22.5 (−13.6 and 10.8), −21.7 to 15.8 (−7.3 and 7.1), and −26.1 to 18.7 (−10.0 and 7.7) mm along the CC (Cranial +), LR (Left +), and AP (Anterior +) directions, respectively. The ranges of the intrafractional wall movements (5% and 95% quantiles) were −5.3 to 14.7 (−2.1 and 4.4), −13.3 to 4.4 (−2.0 and 2.1), and −9.5 to 8.9 (−3.0 and 2.8) mm along the CC, LR, and AP directions. The incidence of sessions requiring realignment due to intrafractional wall movements was 35.9%. The number of realignments in a session after the initial patient setup for all treatments was from 0 to 4, and more than two adjustments were required in 35 sessions with 14 patients. Figure 2 shows the magnitudes of the wall movements in the primary six directions plotted against time before (interfractional) and after (intrafractional) the initial setup at the start of all sessions.

The overall mean uncertainty of position (M), the total systematic uncertainty of position (Σ), and the overall random uncertainty of position (σ) for both inter- and intra-fractional bladder wall movements are shown in Tables 3 and 4.

**Table 3:** Interfractional uncertainty of position (mm) and p-values of the comparison tests

Tumor location group		CC direction			LR direction			AP direction		
		M	Σ	σ	M	Σ	σ	M	Σ	σ
CC group	Cranial	−1.2	4.5	5.0	0.1	3.1	3.1	−1.0	3.5	4.7
	Caudal	−1.0	2.6	6.9	0.9	2.0	4.1	0.0	3.0	4.8
	p-value	0.92	0.09	0.57	0.41	0.20	0.25	0.43	0.44	0.18
LR group	Left	−1.8	4.6	5.1	0.4	3.1	4.0	0.7	3.0	5.0
	Right	−0.5	2.0	7.0	0.7	1.9	3.4	−1.6	3.0	4.5
	p-value	0.36	0.02	0.91	0.78	0.27	0.68	0.05	0.89	0.95
AP group	Anterior	−2.3	3.4	7.4	1.4	2.3	3.4	−1.2	4.3	4.5
	Posterior	−0.4	3.7	5.2	0.0	2.6	3.9	0.0	2.3	4.9
	p-value	0.15	0.80	0.80	0.15	0.95	0.95	0.44	0.04	0.91
Overall		−1.1	3.5	6.1	0.5	2.5	3.7	−0.5	3.2	4.8

*Abbreviations*: *M* mean overall uncertainty of position, *Σ* systematic uncertainty of position, *σ* random uncertainty of position, *CC* cranial-caudal, *LR* left-right, *AP* anterior-posterior

**Table 4:** Intrafractional uncertainty of position (mm) and p-values of the comparison tests

Tumor location group		CC direction			LR direction			AP direction		
		M	Σ	σ	M	Σ	σ	M	Σ	σ
CC group	Cranial	0.8	**2.2**	1.9	0.0	0.6	1.1	-0.4	0.7	2.0
	Caudal	0.0	**0.3**	1.4	-0.1	0.5	1.6	0.1	0.5	1.4
	p-value	0.20	**0.01**	0.44	0.48	0.91	0.47	0.06	0.53	0.24
LR group	Left	0.0	0.4	1.6	0.0	**0.2**	1.3	-0.1	0.5	1.1
	Right	0.7	2.0	1.7	-0.2	**0.7**	1.5	-0.2	0.7	2.1
	p-value	0.22	0.03	0.95	0.32	**0.01**	0.33	0.57	0.82	0.25
AP group	Anterior	1.0	**1.0**	1.9	0.0	0.6	1.3	-0.2	0.2	1.9
	Posterior	0.0	**0.4**	1.5	-0.1	0.5	1.5	-0.1	0.8	1.5
	p-value	0.19	**<0.01**	0.79	0.76	0.76	0.54	0.76	0.09	0.98
Overall		0.4	1.5	1.6	-0.1	0.5	1.4	-0.1	0.6	1.7

*Abbreviations M* mean overall uncertainty of position, *Σ* systematic uncertainty of position, *σ* random uncertainty of position, *CC* cranial-caudal, *LR* left-right, *AP* anterior-posterior

Bold: significant difference between two groups (*p* < 0.05 / 3)

Overall, the interfractional movement of tumors in the cranial tumor groups showed larger Σ than in the caudal tumor groups in the CC direction, and the anterior tumor groups showed larger Σ than the posterior tumor groups in the AP direction. The left-sided tumor groups also showed larger Σ than the right-sided tumor groups in the CC direction, but these differences did not reach significance.

The intrafractional motion showed significantly larger Σ in the CC direction with the cranial and anterior tumor groups. In addition, the right-sided tumor groups showed larger Σ in the LR directions than the left-sided tumor groups.

**Fig. 3:**
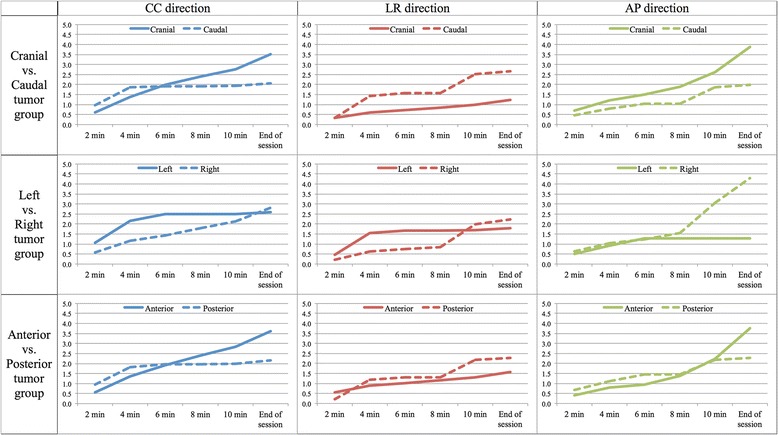
The variances in the intrafractional movement per time segment. The horizontal axis shows the time at 2, 4, 6, 8 and 10 min after the initial setup and the final point is the end of session. The vertical axis represents the variance of the intrafractional bladder wall movement. The variances in the cranial vs. caudal, left vs. right, and anterior vs. posterior tumor groups in the CC, LR, and AP directions are shown together

**Fig. 4:**
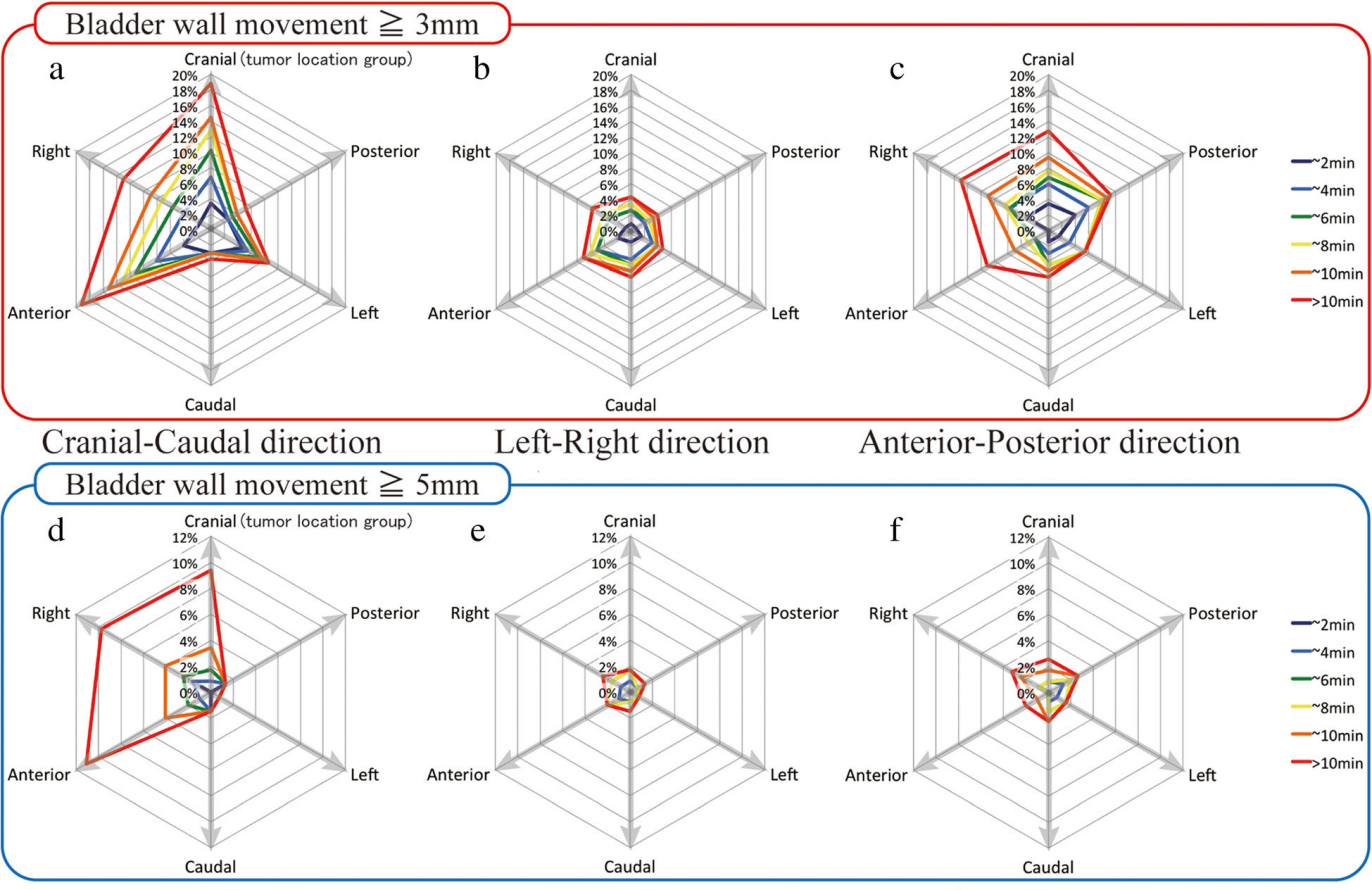
The percentage of the sessions where 3 mm and 5 mm or more of intrafractional wall movements occurred. **a**, **b**, and **c** show the percentage of the sessions that 3 mm or more of intrafractional wall movements within 2(*dark blue*), 4(*blue*), 6(*green*), 8(*yellow*), 10(*orange*) minutes, and after more than 10 min (*red*) for each tumor location group in the Cranial-Caudal, Left-Right, and Anterior-Posterior directions, respectively. Similarly, (D), (E), and (F) show the percentages of sessions with 5 mm or more of intrafractional wall movements in each direction

The variances per time segment are shown in Fig. 3. In general, the variance increased over time, however, the incremental variances of the cranial tumor and anterior tumor group in the CC and AP directions were prominent. The percentage of the sessions where intrafractional wall movements were determined for the cranial vs caudal, left vs right, and anterior vs posterior tumor groups are shown in Fig. 4. In the CC direction, the percentage of the sessions where 3 mm or larger wall movements occurred was increasing over time in a treatment session in patients with cranial, anterior, and right tumors, while there was little or no change with caudal and posterior tumors. Sessions with 5 mm or larger wall movements occurred within 10 min in fewer than 5% of the sessions, but there were steadily increasing wall movements especially in the cranial, anterior, and right tumor groups. The percentages of sessions with 3 mm and 5 mm or larger wall movement in the AP direction was larger than in the LR direction. There were no clear relationships between the tumor location and the percentage of sessions with 3 mm and 5 mm or larger wall movement in the AP and LR direction.

## Discussion

With advances in image guidance technology, CBCT is now commonly used to assess interfractional and intrafractional bladder wall movements. For interfractional bladder movements, a study by Yee et al. reported on daily CBCT for bladder cancer radiotherapy that there were significant large individualized interfractional variations in bladder size and position during a course of radiotherapy [24]. Foroudi et al. reported intrafractional bladder motion estimated from pretreatment and posttreatment CBCT images where the margins are required to cover the intrafractional bladder changes, in mm from pretreatment to posttreatment, were 12.5 (range, 11.9–15.0) in the superior, 6.7 (range, 5.8–11.2) in the inferior, 7.4 (range, 5.9–9.4) in the right, 7.3 (range, 5.1–10.0) in the left, 12.0 (range, 8.5–13.2) in the anterior, and 8.6 (range, 7.3–9.9) in the posterior directions [29]. Foroudi et al. assessed changes of position and volume of the whole bladder between the start and the end of the treatment; however, they could not observe the motion of the tumor-involved bladder wall during the actual treatment. The advantage of the RTRT system is its ability to assess positional changes of the involved bladder wall part continuously during the actual beam delivery.

Our data showed large interfractional bladder wall movements especially in the CC direction and smaller movements in the LR direction. The interfractional uncertainty of position is generally larger than intrafractional uncertainty of positions. It indicates that the large CTV to PTV margins for interfractional bladder movement over-compensate intrafractional movement after the image guided registration. In the intrafractional bladder wall movements, the percentage of sessions where 3 mm or more of wall movements were determined was increasing during the time of the treatment but that of 5 mm or larger wall movements was less than 5% within 10 min. These results are consistent with previous reports. The patients with tumors in the right bladder wall in our series showed a higher incidence of intrafractional wall movements larger than 3 mm in the CC direction than tumors in the left bladder wall. The reasons for this difference are not clear. One possible reason could be that the small bowel or cecum deformed the bladder wall, but further investigation is needed to obtain more details of patient-specific and region-specific bladder wall movements during radiotherapy.

There are several limitations to the study. First, the fiducial markers implanted around the tumor bed were surrogates of the bladder wall and may be different from the motion of the bladder wall itself. Based on our published clinical results using the same registration technique, we believe that the markers represented bladder wall positions well [34]. Second, the number of markers implanted and the registration method was changed in February 2002. If the positional relationship of the markers was remarkably changed from that at the treatment planning CT, the positional relationship between the isocenter and the center of gravity of the three markers or single marker could change. We acquired additional CT scans and confirmed the positional relationship if positional changes in the markers were suspected, however, we believe any consequences of the differences arising from the number of markers was small. Third, this study is retrospective and the patient number is too small to analyze the partial bladder wall movements and the deformation of the bladder in detail. It would be valuable to be able to analyze the possible effect of the bladder volume on the interfractional and intrafractional bladder wall movements and its interplay with the marker positions and also on the anatomical relationships between the markers and other organs, such as bowels and bones during the irradiation.

## Conclusions

Large CTV to PTV margins for interfractional bladder movement might over-compensate intrafractional movement in the image guided radiotherapy era. The cranial and anterior tumors showed larger intrafractional uncertainties of position than the caudal and posterior tumor group especially in the CC direction. A 5 mm internal margin would be sufficient if the treatment finishes within 10 min after the initial image-guided registration, but special attention is recommended for cranial and anterior tumors. Optimal internal margins in each direction should be chosen or a precise intrafractional target localization system is required depending on the tumor location and treatment delivery time in the setting of the partial bladder radiotherapy.

## Abbreviations

3D-RTP: Three-dimensional radiation treatment planning
AP: Anterior-posterior
CBCT: Cone-beam computed tomography
CC: Cranial-caudal
CTV: Clinical target volume
LR: Left-right
MRI: Magnetic resonance imaging
PTV: Planning target volume
RTRT: Real-time tumor-tracking radiotherapy
TUR-BT: Transurethral resection of the bladder tumors
